# Quantification of phosphorus in single cells using synchrotron X-ray fluorescence

**DOI:** 10.1107/S0909049510014020

**Published:** 2010-05-15

**Authors:** Daliángelis R. Núñez-Milland, Stephen B. Baines, Stefan Vogt, Benjamin S. Twining

**Affiliations:** a Department of Chemistry and Biochemistry, University of South Carolina, Columbia, SC 29208, USA; b Department of Ecology and Evolution, Stony Brook University, Stony Brook, NY 11755, USA; c Experimental Facilities Division, Advanced Photon Source, Argonne National Laboratory, Argonne, IL, USA

**Keywords:** phytoplankton, diatom, TEM grid, single-cell analysis, *Thalassiosira pseudonana*

## Abstract

Phosphorus is required for numerous cellular compounds and as a result can serve as a useful proxy for total cell biomass in studies of cell elemental composition. Single-cell analysis by synchrotron X-ray fluorescence (SXRF) enables quantitative and qualitative analyses of cell elemental composition with high elemental sensitivity. Element standards are required to convert measured X-ray fluorescence intensities into element concentrations, but few appropriate standards are available, particularly for the biologically important element P. Empirical P conversion factors derived from other elements contained in certified thin-film standards were used to quantify P in the model diatom *Thalassiosira pseudonana*, and the measured cell quotas were compared with those measured in bulk by spectrophotometry. The mean cellular P quotas quantified with SXRF for cells on Au, Ni and nylon grids using this approach were not significantly different from each other or from those measured spectrophotometrically. Inter-cell variability typical of cell populations was observed. Additionally, the grid substrates were compared for their suitability to P quantification based on the potential for spectral interferences with P. Nylon grids were found to have the lowest background concentrations and limits of detection for P, while background concentrations in Ni and Au grids were 1.8- and 6.3-fold higher. The advantages and disadvantages of each grid type for elemental analysis of individual phytoplankton cells are discussed.

## Abstract 2

Phosphorus abundance was quantified in individual phytoplankton cells by synchrotron X-ray fluorescence and compared with bulk spectrophotometric measurements to confirm accuracy of quantification. Figures of merit for P quantification on three different types of transmission electron microscopy grids are compared to assess possible interferences.

## 1. Introduction

There is significant interest in the concentrations of phosphorus in cells. Phosphorus is required for numerous cellular compounds, including membrane-bound phospholipids, energy-storage molecules such at ATP, and the phosphate backbone of DNA and RNA (Sterner & Elser, 2002 ▶). As a result, P can serve as a useful proxy for total cell biomass in studies of cell elemental composition (*e.g.* Brand, 1991 ▶; Luengen *et al.*, 2007 ▶), and P can be used to identify the location of the nucleus in microprobe studies (Paunesku *et al.*, 2006 ▶). The availability of P often limits the growth of phytoplankton in freshwater systems (Schindler, 1977 ▶; Elser *et al.*, 1990 ▶), as well as in some marine systems (Mills *et al.*, 2004 ▶). Cells can accumulate and store excess P (Diaz *et al.*, 2008 ▶), impacting P biogeochemistry and suggesting that P quotas in cells from natural systems may vary over ecologically relevant temporal and spatial scales.

Synchrotron X-ray fluorescence (SXRF) is a microanalytical technique that enables quantitative and qualitative element analyses of individual cells (Paunesku *et al.*, 2006 ▶). In addition to providing cellular element concentrations, SXRF can produce two-dimensional maps of element distribution of cells with submicrometer resolution (Twining *et al.*, 2003 ▶; Kemner *et al.*, 2004 ▶). SXRF microprobes are being utilized on an increasingly broad range of biological specimens, including elemental imaging of mammalian (Yoshida *et al.*, 2003 ▶) and bacterial (Kemner *et al.*, 2004 ▶) cells and phytoplankton (Twining *et al.*, 2003 ▶). In medicine, SXRF has been used to study metal accumulation in Alzheimer patients’ brain tissue (Miller *et al.*, 2006 ▶) and to quantify elements in the nervous system tissue of patients with neurodegenerative disorders (Yoshida *et al.*, 2003 ▶). Also it has been used to study how infectious pathogens, such as *Mycobacterium tuberculosis*, accumulate metals from host organisms (Wagner *et al.*, 2005 ▶). Because cellular specimens do not require sectioning prior to SXRF, synoptic analyses of biological samples are possible.

Several challenges are associated with the analysis of cellular P using SXRF. Spectral interferences may be introduced by the mounting substrate, although the degree of interference will depend on the energy resolution of the detector. Silicon nitride windows and gold electron microscopy grids can both introduce potential interferences with the P *K*α emission line at 2.014 keV (Si *K*α = 1.740 keV, Au *M*α = 2.123 keV). While other grid materials are available (*e.g.* Al, Ni, Cu), these may cause interferences with metallic analytes of interest. Additionally, appropriate certified reference standards are not readily available for P, and the NIST thin-film standards commonly used for the transition metals (SRM 1832, SRM 1833) do not contain P. In the absence of a certified P standard, researchers have prepared secondary standards with known element stoichiometries (*e.g.* CaHPO_4_) to generate P conversion factors (Twining *et al.*, 2004 ▶); however, the accuracy of this approach has not been directly tested. Further, the preparation and analysis of a separate set of P standards introduces additional laboratory costs and beam time requirements. While artifacts relating to self-absorption are unlikely to be of consequence for cells <20 µm (Twining *et al.*, 2003 ▶), it is uncertain to what degree such standards can be used to infer concentrations in biological samples (Ingram *et al.*, 1999 ▶).

Previously we have confirmed the accuracy of SXRF measurements of Si and the metals Fe, Mn, Ni and Zn in marine and freshwater phytoplankton quantified with the NIST standards (Twining *et al.*, 2003 ▶). Here we test the ability of SXRF to accurately quantify P in individual cells of a model organism (the marine diatom *Thalassiosira pseudonana*) using analytical conversion factors for P empirically derived from the other elements in the NIST thin-film standards. Additionally, we compare P quantification in *T. pseudonana* using three commonly available transmission electron microscopy (TEM) grid materials (gold, nickel and nylon) to test for the influence of potential interferences on P measurements.

## 2. Materials and methods

### 2.1. Culture conditions

Cultures of *T. pseudonana* were grown in enriched seawater media under controlled temperature and light using sterile and trace-metal ‘clean’ techniques. Polycarbonate culture flasks and other plasticware were cleaned by sequential soaks in 1% Micro detergent, 2 *M* reagent-grade HCl and 0.25 *M* trace-metal-grade HNO_3_ with multiple rinses in EPure water (>18.2 MΩ; Barnstead) between each step. Plasticware was dried in a Class-100 hood and stored in clean polyethylene bags until use.

Cells to be mounted on Au or Ni grids were cultured in *f*/50 media (Guillard, 1975 ▶) prepared in 0.22 µm filtered seawater collected from the western North Atlantic Ocean. Cultures were grown at 293 K on a 14:10 h light:dark cycle of 70 µmol photons m^−2^ s^−1^ of cool white light. Parallel cultures were prepared for analysis with SXRF and spectrophotometry. A second culture of cells to be mounted onto nylon grids were cultured in *f*/2 media prepared in the same seawater as above. Both *f*/50 and *f*/2 media provide a P-replete nutrient environment for the cells (1.5 and 36.3 µ*M* phosphate, respectively) compared with concentrations in surface ocean waters. All cultures were sampled during log-phase growth and were diluted to comparable cell concentrations (approximately 40000 cells ml^−1^) prior to mounting on grids. A Z2 Coulter Counter (Beckman Coulter) was used to monitor the concentration of cells in the cultures.

### 2.2. Sample collection

Polyethersulfone membranes (0.45 µm, 47 mm; Supor 450, Pall) were used to collect cells for bulk P analyses. Membranes were soaked in 1 *M* HCl at 333 K for 24 h and rinsed copiously with EPure water prior to use. For the bulk samples, approximately 500 ml of the culture was filtered onto acid-cleaned membranes and then frozen at 253 K until analysis. Samples for SXRF analysis were prepared following the procedures of Twining *et al.* (2003 ▶). Aliquots of the cultures were preserved using glutaraldehyde to a final concentration of 0.25%. The samples were mounted on Au, Ni or nylon TEM grids coated with C/Formvar film. The Au and Ni grids were purchased from Electron Microscopy Sciences pre-coated with film, and the nylon grids were coated manually in the laboratory. The cells were centrifuged onto the grids (3345 × *g*) in polyethylene tubes. The supernatant was gently decanted, and the grids were immediately removed with Teflon-coated forceps. The grids were briefly rinsed with EPure water to remove salt residue and dried in a darkened Class-100 clean bench. After 15–20 min the grids were observed using light microscopy and then stored under dark conditions until SXRF imaging.

### 2.3. Element analyses

Cells collected on membranes were analyzed using a method adapted from Aspila *et al.* (1976 ▶). Membranes were weighed, cut in half and each portion re-weighed. Filter pieces were placed in acid-cleaned glass vials and combusted for 4.5 h at 773 K.  Following combustion, P was extracted from each filter sample with 10 ml of 1.2 *M* HCl for 2.5 h. The samples were centrifuged and the extract diluted ten-fold with EPure water.  Samples were analyzed for soluble reactive P using a colorimetric technique on a UV/VIS spectrophotometer (Beckman DU640) at 880 nm (Koroleff, 1983 ▶). Recovery and accuracy were checked both with P spikes added to samples and with NIST standard reference material made of tomato leaves (NIST SRM 1573a).

Cells mounted onto TEM grids were analyzed with SXRF at the 2-ID-E hard X-ray microprobe during two trips to the Advanced Photon Source at Argonne National Laboratory. An incident beam tuned to 10 keV was used to stimulate *K*-line emissions for the elements with atomic numbers ranging from Si through Zn. The beam was focused with a Fresnel zone plate to approximately 300 nm. Target cells were scanned through the focused beam, and the entire fluorescence spectrum was recorded at each pixel using an energy-dispersive Ultra LEGe detector (Canberra, Meriden, CT) with a 100 mm^2^ sensitive area and a 24 µm Be window. The pixel step size was 0.2 µm and detector dwell times were 0.5–1 s. On the first trip (November 2007), 20 and 21 cells mounted on Au and Ni grids, respectively, were analyzed. During the second trip (April 2008), seven cells on nylon grids were analyzed.

### 2.4. Data analysis

The spectra from each pixel in the cell were summed to generate a single spectrum for each cell, and this spectrum was corrected for instrumentation background using an ‘empty’ background region next to the cell. The cellular and background spectra were analyzed using MAPS software designed specifically for this beamline (Vogt, 2003 ▶). The cellular Si, K, Ca, Ti, V, Mn, Fe, Co, Cu and Zn concentrations were calculated from peak areas by comparison with NIST thin-film standards (SRM 1832, SRM 1833).

Calibration coefficients for P (as well as S and Ni) were calculated empirically from linear regressions of measured fluorescence yield against theoretical fluorescence yield [taken from Bearden (1967 ▶)]. Calibration coefficients used to calculate elemental concentrations were then averaged over the course of each run (∼four analyses). Limits of detection were calculated for each grid type for the elements Si, P, Mn, Fe, Co, Ni, Cu and Zn. Limits of detection were calculated as three times the standard deviation of the averaged background region of interest, an area outside but adjacent to the cell.

## 3. Results and discussion

### 3.1. Calculation of P calibration coefficients

**Figure 1:**
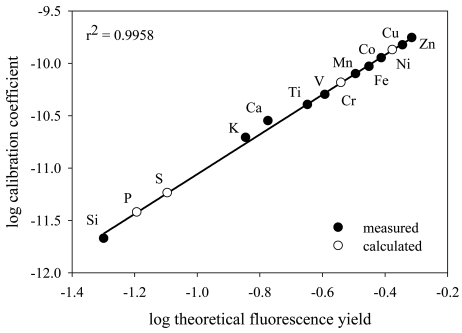
Measured elemental calibration coefficients plotted against the theoretical fluorescence yield. The ordinate units are counts (µg cm^−2^)^−1^ s^−1^ pixel^−1^ DS_IC^−1^. Theoretical fluorescence yields were obtained from Bearden (1967 ▶). The closed circles denote the measured calibration coefficients and the open circles show the coefficients calculated using the regression equation.

Calibration coefficients for P, S and Ni were calculated empirically by interpolation between calibration coefficients of elements in the NIST thin-film standards. Fig. 1 ▶ shows a typical relationship of the measured calibration coefficients plotted against the theoretical fluorescence yield for the elements Si, K, Ca, Ti, V, Mn, Fe, Co, Cu and Zn. Measured fluorescence counts are normalized to element concentration, detector dwell time, number of pixels and the flux of X-ray photons downstream of the sample. There is a tight linear relationship between these variables when plotted in log space (*y* = 1.898*x* − 9.161, *r* ^2^ = 0.9958). From these relationships the calibration coefficients for P, S and Ni were interpolated for each run. Phosphorus calibration coefficients calculated from separate standards analyzed over the course of a four-day run varied by only 5%, indicative of adequate normalization procedures, stable beam conditions and analytical sensitivity during each run.

The P conversion factors calculated in this manner vary from those calculated previously using dried inorganic salt solutions (Twining *et al.*, 2003 ▶). In that approach CaHPO_4_ was dissolved in a dilute HCl solution, dropped onto polycarbonate membranes and allowed to dry in a trace-metal clean laminar flow hood. The concentration of Ca in the dried material was determined with SXRF based on the calibration coefficients obtained from the NIST standard. The concentration of P was estimated from the Ca assuming a molar ratio of 1:1 and the calibration coefficient determined by dividing this number by the observed count rate of fluorescent photons after correcting for incident flux. This approach resulted in a P calibration coefficient of 6.06 × 10^−8^ counts (µg cm^−2^)^−1^ s^−1^ pixel^−1^ DS_IC^−1^, which was approximately two-fold less than the value of 1.34 × 10^−7^ counts (µg cm^−2^)^−1^ s^−1^ pixel^−1^ DS_IC^−1^ calculated in this study (where DS_IC refers to counts per second in an ionization chamber located immediately downstream of the sample).

This difference results in an equivalent variation in cellular P quotas calculated using these two approaches, with the regression approach resulting in higher apparent analytical sensitivity for P and therefore lower calculated cellular P quotas. This discrepancy could indicate that a large fraction of P fluorescence photons were absorbed by the matrix of the calcium phosphate granules that we measured. As a first-order approximation of the sample mass thickness (µg cm^−2^) through which fluorescence photons emitted by P must pass, we used our initial measurements of the areal concentrations of P, Cl and Ca. We obtained attenuation constants of 296, 441 and 770 cm g^−1^ at the P *K*α emission edge for P, Cl and Ca, respectively, from the NIST tables of X-ray mass attenuation coefficients and mass energy-absorption coefficients. Given these coefficients, we estimated the fraction of P fluorescence photons that would be absorbed by the standard matrix assuming that the average photon must pass through half the sample on average before reaching the detector (*i.e.* that fluorescence photons originated evenly throughout the sample). The total attenuation of fluorescence photons from P amounted to <6%, which is not enough to cause a two-fold discrepancy between measured and real P fluorescence.

Living cells were far less prone to absorb P fluorescence photons because they were less optically thick. The mass thickness of P and Ca in standards ranged from 27–33 µg cm^−2^ and 74–90 µg cm^−2^, respectively. The maximum values for cells measured on the same run were 4.85 µg cm^−2^ P and 1.9 µg cm^−2^ Ca, respectively, while the average values were 0.51 µg cm^−2^ P and 1.9 µg cm^−2^ Ca. Organic matter can also contribute to absorbance of fluorescence photons. However, C and O have attenuation constants at the P *K*α edge of 300 and 700 cm g^−1^, both of which are lower than the attenuation constants of Ca, and only in the largest cells does the mass thickness of C or O approach that of Ca in the standards.

Instead, the molar ratio of Ca and P in the granules analyzed must have differed substantially from the 1:1 ratio in the standard solution. The Ca:P molar ratio in calcium phosphate minerals can vary substantially: 5:3 for hydroxylapatite, 3:2 for tricalcium phosphate, 1:1 for calcium monohydrogen phosphate, and 1:2 for calcium dihydrogen phosphate. In addition, Cl^−^ present in the dilute acid solution could have combined with Ca^2+^ to form CaCl_2_ or become incorporated into apatite. As the standard solution dried, the least soluble forms, apatite and tricalcium phosphate, would have precipitated first, followed by calcium monohydrogen phosphate, then calcium dyhydrogen phosphate and, finally, calcium chloride. Since the standard solution dried from the outside toward the center of the droplet, the Ca:P stoichiometry of precipitates in different parts of the filter probably vary significantly from the 1:1 molar ratio that must have applied across the filter as a whole. The granules we analyzed to determine the P calibration coefficient were located near the periphery of the area of precipitate deposition on this filter. Only in this region were the precipitate granules of a size that produced a fluorescence signal small enough not to overload the detectors yet large enough to ensure rapid quantification. As these granules were probably among the first to form, they are more likely to be comprised of the least-soluble forms of calcium phosphate. Indeed, after correcting for self-absorption of fluorescent photons of all elements, the average Ca:P:Cl stoichiometry for all elements was 1:0.56:0.32. This stoichiometry is consistent with either pure chlorapatite (1:0.6:0.2) or a 2:1 molar ratio of tricalcium phosphate (3:2:0) and calcium chloride (1:0:2).

Our results suggest that caution must be used when preparing standards for SXRF analyses. As has been observed before, stoichiometric assumptions that should be valid over centimeter and millimeter scales are not necessarily valid at submicrometer scales. This caveat is especially appropriate when producing standards using known solutions from which multiple minerals may precipitate. On the other hand, when only one precipitate is possible, it should be possible to make standards to cross-check the indirect method of determining calibration coefficients employed here. For example, standards made by drying a solution of zinc sulfate onto membrane filters yielded a molar ratio of Zn:S (1.02 ± 0.04) that was indistinguishable from the expected ratio of 1:1 calculated from the interpolated S calibration coefficient. Therefore, we believe that past estimates of S in cells that were based on these standards are correct.

### 3.2. Phosphorus contents of *T. pseudonana*

**Figure 2:**
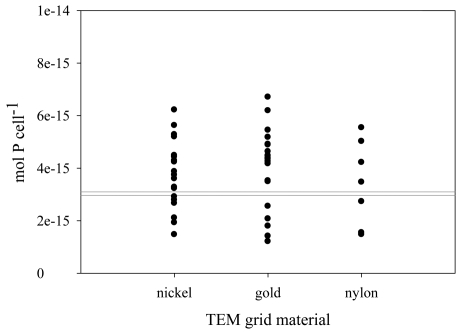
Scatter plot of cellular P quotas in *T. pseudonana* mounted on three different grid materials as measured with SXRF. The quota (mol P cell^−1^) measured in each individual cell is shown. The gray lines demarcate the upper and lower range of cell quotas measured for the same cultures with bulk spectrophotometry.

The accuracy of the derived P calibration coefficients was assessed through measurements of this element in cultured cells of the marine centric diatom *T. pseudonana*. This is the first diatom with a sequenced genome (Armbrust *et al.*, 2004 ▶), and it is a common model organism for laboratory studies of phytoplankton physiology and nutrient requirements (Sunda *et al.*, 1991 ▶; Baines *et al.*, 2001 ▶). Between 7 and 21 cells were analyzed for each grid type during two separate trips to the Advanced Photon Source. A total of 48 *T. pseudonana* cells were analyzed, and the P quotas are presented in Table 1 ▶ and Fig. 2 ▶. Notable intra-population variability was observed. Phosphorus quotas ranged 4.2-, 5.0- and 3.8-fold for cells on the Au, Ni and nylon grids, respectively, resulting in coefficients of variability (CV) between 38 and 47%. Some variation among cells is expected. For example, cells must essentially double their nucleic acid content in the interval between cell divisions, so P content may reflect the cell’s place in the cell cycle. Also, cell size can vary among diatom cells because the architecture and rigidity of the diatom shell results in a continuous reduction in cell size through the generations. Researchers using other single-cell analytical techniques to measure P, C, N and S in individual phytoplankton cells have observed similar degrees of intra-population variability to those reported here. Gisselson *et al.* (2001 ▶) used a proton microprobe to study P quotas of *Dinophysis norvegica* collected from the Baltic Sea and reported a CV of 32% among individual dinoflagellate cells. They also report CVs of 23% and 49% for C and N, respectively. Heldal *et al.* (2003 ▶) analyzed C, N, O, P and S in two groups of cultured cyanobacteria, *Prochlorococcus* and *Synechococcus*, using an electron microprobe. Resulting CVs within these populations varied from 23 to 81%. The analytical precision of SXRF, assessed through replicate analyses of individual cells, has been shown to be better than 10% (Twining *et al.*, 2003 ▶), so the range of *T. pseudonana* P quotas measured here is therefore real and unlikely to be explained by a lack of analytical precision.

The mean P quotas of *T. pseudonana* mounted on either Au, Ni or nylon grids were 4.0 × 10^−15^, 3.8 × 10^−15^ and 3.4 × 10^−15^ mol cell^−1^, respectively (Table 1 ▶). The mean P quotas were not significantly different among the three grid types (One-way ANOVA, *p* = 0.65). The mean (± SD) quota of *T. pseudonana* collected on triplicate filters was 3.0 × 10^−15^ ± 7.0 × 10^−17^ mol P cell^−1^ (Table 2 ▶). This value was not significantly different from the mean quotas for cells mounted on grids (Wilcoxon test, *p* = 0.24). The SXRF P quotas were also compared with the results of other researchers using independent techniques to assess the biomass of *T. pseudonana*. Sunda & Huntsman (1995 ▶) measured cellular C in *T. pseudonana* grown over a range of Fe conditions, and the C quotas were converted to P using the Redfield ratio of 106 C:P (Redfield, 1958 ▶; Redfield *et al.*, 1963 ▶) producing a range of P quotas from 2.8 × 10^−15^ to 6.1 × 10^−15^ mol cell^−1^ (Table 2 ▶). This range spans the mean quotas measured with SXRF, as well as that measured on bulk samples with spectrophotometry. Thompson *et al.* (1992 ▶) measured cellular N and C quotas in *T. pseudonana*, and these were converted to P quotas using the Redfield ratio of 106C:16N:1P (Redfield *et al.*, 1963 ▶). The range of P quotas derived from Thompson *et al.* (1992 ▶) in this manner (6.2 × 10^−15^–7.8 × 10^−15^ mol cell^−1^) is also comparable with those quantified by SXRF in this study (Table 2 ▶). Use of previously analyzed calcium phosphate standards for P quantification results in a mean quota of 8.9 × 10^−15^ mol cell^−1^, twice the P quota obtained using the bulk analytical technique. This study provides additional support for the accuracy of P quantification in phytoplankton by SXRF using conversion coefficients calculated from those of other elements in the NIST standards and confirms that the NIST standards are representative of biological samples.

### 3.3. Comparison of grid types

**Figure 3:**
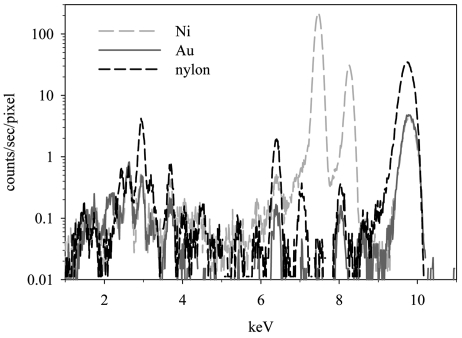
Spectra of background regions in three types of C/Formvar-coated TEM grids.

Several grid substrates were compared for their suitability to P quantification in phytoplankton. Gold grids are commonly used in SXRF analyses. However, because of the potential interference of the Au *M*α line with the P *K*α emission lines, Ni and nylon grids were evaluated as alternative substrates. Differences were observed in the apparent background P concentrations on the grids, which were nearly three-fold higher in Au grids (1.7 × 10^−2^ µg cm^−2^) than in Ni grids (5.9 × 10^−3^ µg cm^−2^) (Table 1 ▶, Fig. 3 ▶). Even lower P background concentrations (4.4 × 10^−3^ µg cm^−2^) were measured in the nylon grids. Despite modeling of the Au *M*α peak at 2.123 keV with MAPS, Au fluorescence can obscure the modeled signal from the P *K*α peak at 2.014 keV when the beam is close to the Au grid bar and the Au signal is very high relative to the P signal. These same factors contribute to the higher P detection limits measured for Au grids. The limit of detection for P in cells of this size and under these analytical conditions was six times higher for Au grids (5.6 × 10^−16^ mol cell^−1^) than Ni grids (8.7 × 10^−17^ mol cell^−1^) and 26 times higher than nylon grids (2.1 × 10^−17^ mol cell^−1^) (Table 1 ▶). Nylon grids thus appear to be optimal for measurements of P in phytoplankton cells, owing to lower detection limits and lower apparent P background concentrations. However, nylon grids bring added difficulties owing to their static charging nature, which complicates handling of the grids. In addition, nylon grids do not provide a smooth substrate, and they have no reference point, potentially complicating sample targeting. Thus choice of grid material needs to be made within the context of the samples to be collected and the elemental analytes of interest.

**Figure 4:**
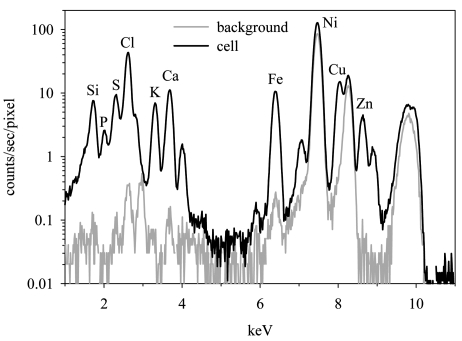
Spectra of the cell and an adjacent empty background region for a marine diatom cell mounted on a C/Formvar-coated Ni TEM grid. The *K*α emission lines for the major bioactive elements are labeled.

Phosphorus is often used as a normalizing variable in studies of trace-element contents of natural algae and protozoa (Twining *et al.*, 2004 ▶). Thus the choice of a grid substrate should not only be optimized for estimation of P, but also the other elements of analytical interest. Not surprisingly, the background concentration of Ni in the Ni grids is approximately four orders of magnitude higher (6.3 × 10^−1^ µg cm^−2^) relative to those in Au grids (5.9 × 10^−5^ µg cm^−2^) and nylon grids (1.5 × 10^−5^ µg cm^−2^) (Fig. 3 ▶), and cellular limits of detection for Ni are three to four orders of magnitude higher for Ni grids (9.4 × 10^−14^ mol cell^−1^). This will severely constrain measurements of Ni on Ni grids. Indeed, Ni *K*α fluorescence peaks in *T. pseudonana* cells mounted on Ni grids are only slightly higher than Ni peaks corresponding to adjacent empty background regions (Fig. 4 ▶). Furthermore, measured cellular Ni quotas were approximately 250 times higher in cells mounted on Ni grids than in cells on the other grids, indicating either substantial contamination of the cells during the mounting process, grid fluorescence from scattered X-rays, or both. Therefore, studies in which Ni is an analyte of interest should avoid these grids. Studies of Al, Ti, Cu or Au could also be compromised by the use of a matching grid material.

In summary, P quotas were successfully quantified in the diatom *T. pseudonana* with SXRF using empirical P conversion factors derived from other elements in NIST thin-film standards. Quantification performed in this manner was found to be more accurate than that conducted using separate dried salt standards. Indeed, this study demonstrates that conversion factors can be accurately calculated without dedicating valuable beam time to analysis of separate P standards. Nylon grids were found to have the lowest background concentrations and limits of detection for P; however, Au and Ni grids may also be appropriate, depending on the P content of the target cells and the metal analytes of interest. SXRF is an ideal tool for studying spatial and taxonomic variations in the P quotas of individual cells, and application of this method will lead to advances in our understanding of element dynamics within cellular systems.

## Figures and Tables

**Table 1:** Limits of detection, background quotas and cellular phosphorus quotas of *Thalassiosira pseudonana* as measured by SXRF ‘Run date’ refers to the date when the cells were analyzed by SXRF. The P quotas (mol P cell^−1^) are arithmetic means.

Run date	Grid type	*n*	Limit of detection (µg cm^−2^)	Limit of detection (mol cell^−1^)	P quota in background (µg cm^−2^)	P quota in background (mol cell^−1^)	P quota (mol cell^−1^)	Standard deviation
Nov 2007	Au	20	4.4 × 10^−2^	5.6 × 10^−16^	1.7 × 10^−2^	2.0 × 10^−16^	4.0 × 10^−15^	1.5 × 10^−15^
Nov 2007	Ni	21	7.5 × 10^−3^	8.7 × 10^−17^	5.9 × 10^−3^	5.9 × 10^−17^	3.8 × 10^−15^	1.2 × 10^−15^
Apr 2008	Nylon	7	2.3 × 10^−3^	2.7 × 10^−17^	4.4 × 10^−3^	3.3 × 10^−17^	3.4 × 10^−15^	1.6 × 10^−15^

**Table 2:** Phosphorus quotas (mol P cell^−1^) of *Thalassiosira pseudonana* as measured on individual cells with SXRF and on bulk samples by colorimetry and radioisotopes The quotas are means (± SD) of 48 cells for SXRF and three filters for the colorimetric analyses. The range of quotas measured by Sunda & Huntsman (1995 ▶) were calculated from C measured over a range of Fe concentrations assuming the Redfield ratio of 106:1 C:P. The range of quotas measured by Thompson *et al.* (1992 ▶) was calculated from measurements of cellular C and N using the Redfield ratio of 106C:16N:1P.

SXRF	3.8 × 10^−15^ ± 1.4 × 10^−15^
Spectrophotometric	3.0 × 10^−15^ ± 7.0 × 10^−17^
Sunda & Huntsman (1995 ▶)	2.8 × 10^−15^–6.1 × 10^−15^
Thompson *et al.* (1992 ▶)	6.2 × 10^−15^–7.8 × 10^−15^
